# Specialized Information Processing Deficits and Distinct Metabolomic Profiles Following TM-Domain Disruption of *Nrg1*

**DOI:** 10.1093/schbul/sbw189

**Published:** 2017-03-11

**Authors:** Colm M. P O’Tuathaigh, Naina Mathur, Matthew J O’Callaghan, Lynsey MacIntyre, Richard Harvey, Donna Lai, John L Waddington, Benjamin S Pickard, David G Watson, Paula M Moran

**Affiliations:** 1 School of Medicine, University College Cork, Cork, Ireland;; 2 School of Psychology, University of Nottingham, Nottingham, UK;; 3 Strathclyde Institute of Pharmacy and Biomedical Sciences, University of Strathclyde, Glasgow, UK;; 4 Victor Chang Cardiac Research Institute, Sydney, Australia;; 5 Molecular and Cellular Therapeutics, Royal College of Surgeons in Ireland, Dublin, Ireland;; 6 Jiangsu Key Laboratory of Translational Research & Therapy for Neuro-Psychiatric-Disorders and Department of Pharmacology, College of Pharmaceutical Sciences, Soochow University, Suzhou, China

**Keywords:** mutant phenotype, cognition, metabolome, antipsychotics, neuregulin, prepulse inhibition, choline, lipids, schizophrenia

## Abstract

Although there is considerable genetic and pathologic evidence for an association between neuregulin 1 (*NRG1*) dysregulation and schizophrenia, the underlying molecular and cellular mechanisms remain unclear. Mutant mice containing disruption of the transmembrane (TM) domain of the *NRG1* gene constitute a heuristic model for dysregulation of NRG1-ErbB4 signaling in schizophrenia. The present study focused on hitherto uncharacterized information processing phenotypes in this mutant line. Using a mass spectrometry-based metabolomics approach, we also quantified levels of unique metabolites in brain. Across 2 different sites and protocols, *Nrg1* mutants demonstrated deficits in prepulse inhibition, a measure of sensorimotor gating, that is, disrupted in schizophrenia; these deficits were partially reversed by acute treatment with second, but not first-, generation antipsychotic drugs. However, *Nrg1* mutants did not show a specific deficit in latent inhibition, a measure of selective attention that is also disrupted in schizophrenia. In contrast, in a “*what–where–when*” object recognition memory task, *Nrg1* mutants displayed sex-specific (males only) disruption of “*what–when*” performance, indicative of impaired temporal aspects of episodic memory. Differential metabolomic profiling revealed that these behavioral phenotypes were accompanied, most prominently, by alterations in lipid metabolism pathways. This study is the first to associate these novel physiological mechanisms, previously independently identified as being abnormal in schizophrenia, with disruption of NRG1 function. These data suggest novel mechanisms by which compromised neuregulin function from birth might lead to schizophrenia-relevant behavioral changes in adulthood.

## Introduction

Neuregulin-1 (*NRG1*) is a gene that has been associated with increased risk for schizophrenia across diverse populations.^1–3^ Studies on postmortem brain and serum from schizophrenia cases have reported up-regulation of specific *NRG1/ErbB4* splice variants and increased NRG1 signaling,^4–9^ and decreased isoform-specific expression of NRG1 transcripts.^9,10^ However, despite such combined genetic and pathologic evidence for NRG1 dysregulation in schizophrenia, the mechanisms underlying this association remain unclear.^11^

Multiple NRG1 isoforms have been described, the diversity of which is due to alternative splicing and the existence of multiple 5′ flanking regulatory elements. NRG1 I–III, share the EGF–like signaling domain; interaction of these EGF-like domains with membrane-associated tyrosine kinases (ErbB receptors) activates intracellular signaling pathways.^12–14^ NRG1/ErbB4 signaling has been associated with various neurodevelopmental and plasticity-related processes, including synapse formation, neuronal migration, and neurotransmitter receptor development and function.^13^ Altered NRG1 expression has been identified in several schizophrenia-relevant rodent models,^15–17^ and several *Nrg1* knockout/transgenic mouse lines have been developed to study the impact of altered NRG1 signaling on endophenotypes relevant to schizophrenia.^18,19^ The most well-characterized *Nrg1* mutant model involves heterozygous deletion of the transmembrane domain of the *Nrg1* gene; this model demonstrates behavioral and cellular phenotypes related to schizophrenia, as well as disturbance in schizophrenia-relevant (dopaminergic, GABAergic, glutamatergic) neurotransmission.^19,20^ Recent work has suggested that the TM-domain *Nrg1* mutant is not a straightforward model of NRG1 haploinsufficiency, but may be better characterized as a model of imbalanced NRG1-ErbB4 signaling.^21^

Cognitive dysfunction is highly prevalent in schizophrenia, and there is consensus that impairments in working memory, executive function, and attention are core features.^22^ These cognitive impairments are of particular significance for the disease, as they are more closely associated with poor outcome in patients.^23,24^ Prepulse inhibition (PPI) and latent inhibition (LI) measure sensory gating and selective attention processes, respectively. Patients with schizophrenia show disrupted PPI and LI task performance, and these deficits are considered to reflect aberrant salience processing, that is, at the interface of psychotic symptoms and cognitive dysfunction.^25–30^ Patients with schizophrenia also show profound impairment in episodic memory,^31,32^ which refers to a cross-modal form of memory that encompasses not only the memory for an event (“what”), but also its spatial (“where”) and temporal (“when”) characteristics.^33^ TM-domain *Nrg1* mutants demonstrate intact spatial learning and working memory,^34^ but subtle deficits in contextual fear conditioning, cued aversion, and novel object recognition.^35,36^ While disruption to PPI has been reported, though in a manner highly sensitive to protocol and stress-related environmental factors,^18,19,30,37^ episodic memory and detailed examination of preattentional and selective attention processing has not been conducted in the TM-domain *Nrg1* mutant line.

The relative failure to develop novel antipsychotic drugs in recent decades reflects, in part, incomplete understanding of disease mechanisms, and the absence of treatment biomarkers.^38^ Metabolomic analysis, which provides a snapshot of the current status of biochemical pathways, can provide additional information on pathways affected in disease pathobiology.^39,40^ For example, metabolomic characterization of brain tissue from mice containing a knockout of the *Npas3* gene, which has been independently associated with increased risk for schizophrenia,^41,42^ has revealed differential expression of glycolysis pathway metabolites.^39^

In the following study, we examined whether TM-domain *Nrg1* mutant mice show abnormalities in performance measures of LI, PPI, and an episodic memory (“*what–where–when*”) paradigm. Because of controversies regarding PPI disruption in this mutant line,^18,19,30^ we also examined whether acute treatment with selected first- or second-generation antipsychotic drugs was able to ameliorate PPI deficits. We then investigated the underlying neurobiology of *Nrg1* mutants via high-resolution mass-spectrometry-based metabolomic analysis of brain tissue.

## Methods

### Ethics Statement

All mouse studies conducted in University of Nottingham, UK, were performed in accordance with local and national rules on animal experimentation, and with appropriate personal and project license authority under the Animals (Scientific Procedures) Act, UK 1986 (PPL no: 40/2883). All mouse studies conducted at RCSI, Dublin, were approved by the Research Ethics Committee of RCSI, and were conducted under license from the Department of Health and Children in accordance with Irish legislation and the European Communities Council Directive 86/609/EEC for the care and use of experimental animals.

### Animals

Mice with heterozygous transmembrane-domain deletion of *Nrg1* were originally generated at the Victor Chang Cardiac Research Institute, University of New South Wales, Australia, as described previously.^34,43^ The TM-domain *Nrg1* mutant line was maintained on a C57BL/6 background, with experimental animals generated from heterozygous breeding pairs and genotyped using PCR.

In this report, 2 cohorts of TM-domain *Nrg1* mutants were used, each tested at different phenotyping facilities. Both groups of experimental animals were bred and weaned at the Biomedical Research Facility at RCSI, Dublin: mice used in the LI, PPI (without drug treatment), and episodic memory studies were shipped to the housing facility at the University of Nottingham at 7–10 weeks of age, with testing commencing 3 weeks after arrival; mice used in the PPI study with drug treatment were tested at RCSI, Dublin. In both facilities, mice were housed in groups of 3–5 per cage and maintained at 21°C on a 12-hour light/dark cycle (lights on 08:00 hours), with food and water available ad libitum. At Nottingham Mice were housed in individually ventilated cages (IVC) (Tecniplast) in cages with a floor area 501 sq cm, with the following cage dimensions: 391 mm L × 199 mm W × 160 mm H. Cages had a play tunnel and shredded paper nesting material. At the Dublin facility, mice were housed in IVC cages (Animal Care Systems) in cages with a floor area of 535 sq cm, with the following cage dimensions: 389 mm L × 198 mm W × 241 mm H. Shredded paper nesting material was added.

### Experimental Design

LI, PPI (Nottingham), and episodic memory testing, in that order, were carried out in adult (10–12 weeks) male and female WT (*n* = 16; 8 male, 8 female) and *Nrg1* mutants (*n* = 15; 7 male, 8 female). In the study examining the effects of antipsychotic treatment on PPI (Dublin), adult male mice of each genotypes (8–12 per treatment condition, 16–20 weeks) were treated with either vehicle (WT, *n* = 9; *Nrg1*, *n* = 15), haloperidol (0.5 mg/kg; WT, *n* = 7; *NRG1*, *n* = 7), clozapine (1 mg/kg; WT, *n* = 7; *Nrg1*, *n* = 9), or amisulpride (5 mg/kg; WT, *n* = 9; *Nrg1*, *n* = 7). Thirty minutes later, all mice underwent PPI testing. Metabolomic analysis was carried out in whole brains of male WT (*n* = 4) and *Nrg1* mutants (*n* = 4). As previous studies have indicated significant sex effects on behavioral parameters in these and related mice,^34,44^ we have presented data split by sex for comparative purposes. We were unable to include site as an ANOVA variable as one cohort received injections (Dublin) while the other did not (Nottingham).

Behavioral and psychopharmacological protocols, as well as the metabolomics methods are available in the online supplementary material.

### Data Analysis

Statistical analysis of behavioral and psychopharmacological data was performed using SPSS v.20 (SPSS, Inc.). For all measures, independent or repeated measures analysis of variance (ANOVA) was used with main factors of genotype (*Nrg1* mutant/WT), and sex (male/female). Further information regarding statistical methods, including those used for metabolomics analyses, is available in the online supplementary material.

## Results

### Latent Inhibition Performance in TM-Domain Nrg1 Mutants

**Fig. 1.**
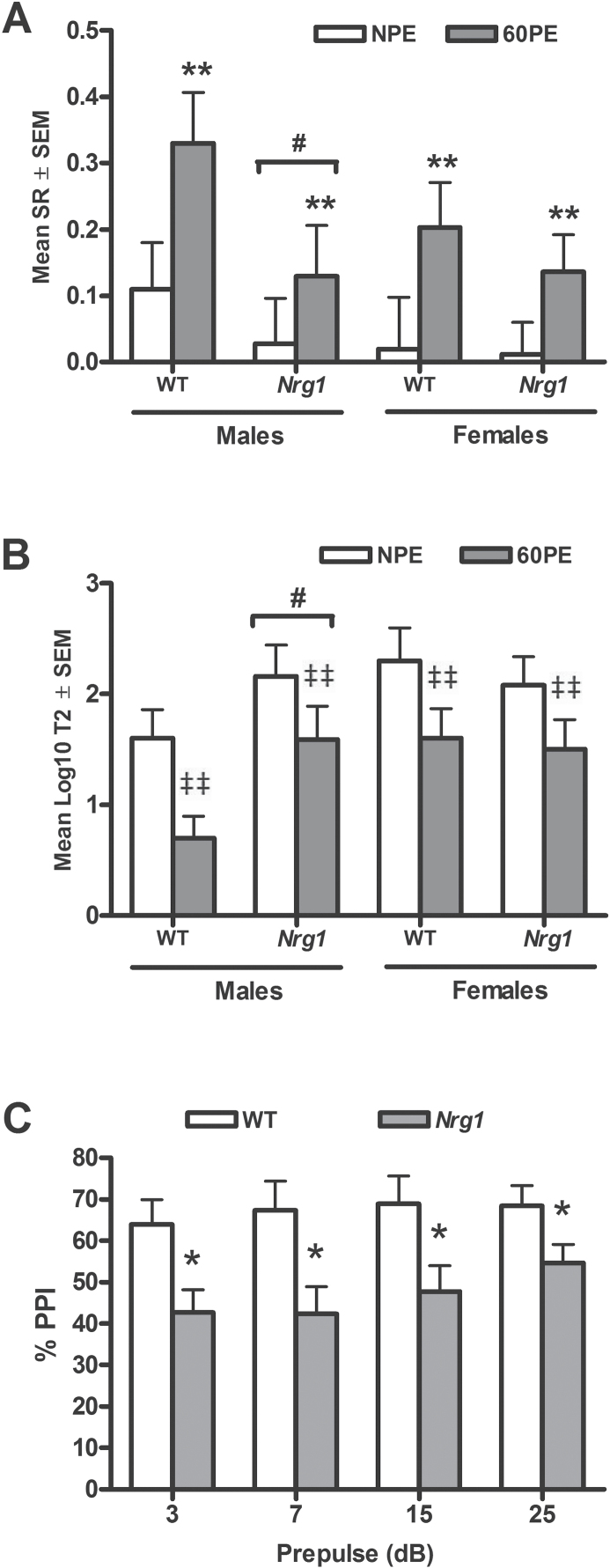
Latent inhibition (LI) and pre-pulse inhibition (PPI) in *Nrg1* mutants. (A) A significant effect of pre-exposure (ie, non–pre-exposed [NPE] vs pre-exposed [PE]) on the suppression ratio (SR) was observed (*P* < .01); **P < .01 vs NPE. No genotype × exposure interaction was observed. Split by sex, male *Nrg1* displayed lower SR values relative to wild-type (WT) controls (*P* < .05); ^#^*P* < .05 vs male WT. No other effects of sex, genotype × sex, or genotype × sex × exposure interaction effects were observed. Data are mean SR ± SEM. (B) Based on log10 transformation of T2 values (time in seconds in NPE and PE conditions taken to resume licking after conditioned stimulus [CS] onset), a significant effect of exposure on log10 T2 times was observed (*P* < .01); ^‡‡^*P* < .01 vs NPE. A significant genotype × sex effect was observed, indicative of increased T2 times across both exposure conditions in male *Nrg1* mutants relative to WT (*P* < .05); ^#^*P* < .05 vs male WT. Data are mean log10-transformed T2 values ± SEM. (C) % PPI is significantly decreased in *Nrg1* mutants vs WT at a pulse intensity of 120 dB and 4 pre-pulse intensity levels (3, 7, 15, and 25 dB above background) (*P* < .05); *P < .05 vs WT. These data were collected at University of Nottingham (see online supplementary material). No genotype × prepulse intensity or genotype × sex interaction effect was observed. Data are mean % PPI ± SEM.

Disrupted LI has been demonstrated in various mutant mouse models of schizophrenia.^45,46^ LI was assessed using a conditioned lick suppression paradigm, where intact LI comprises reduced learning of a conditioned stimulus (CS)–unconditioned stimulus (US) association in a group pre-exposed to that stimulus without reinforcement (pre-exposed, PE) compared with a group without such pre-exposure (non–pre-exposed, NPE).^45^ A significant effect of pre-exposure was observed on suppression ratio (SR) values (*F*_1,54_ = 11.96, *P* < .01), in a manner indicative of intact LI. However, LI was not modified by genotype (*F*_1,54_ = 3.04, *P* > .05), sex (*F*_1,54_ = 1.53, *P* > .05), or any interaction between these factors (*F*_1,54_ = 0.12, *P* > .05; figure 1A). Further analysis is shown in the supplementary data section.

An alternative approach to measuring conditioned lick suppression in the LI paradigm is to express it as the log10 of the T2 values based on time (seconds) taken to resume licking after CS onset in the NPE and PE conditions.^45^ Pre-exposure significantly reduced the time taken to complete licks (log10 times) (*F*_1,54_ = 11.64, *P* < .01), demonstrating that LI was also observed employing this alternative index. No significant effect of genotype (*F*_1,54_ = 2.11, *P* > .05) was observed but the analysis of SR data revealed that male mice, and particularly male *Nrg1* mutants, demonstrated an overall reduction in log10 times (sex, *F*_1,54_ = 4.14, *P* < .05; genotype × sex, *F*_1,54_ = 5.03, *P* < .05; figure 1B). However, they demonstrated no LI deficit, as evidenced by the absence of any significant genotype by exposure by sex interaction (*F*_1,54_ = 0.89, *P* > .05).

### Prepulse Inhibition Performance in TM-Domain Nrg1 Mutants

These PPI data were collected at the University of Nottingham, Nottingham, UK (see Methods and online supplementary material for study design and methodology).

#### Startle Response/Habituation.

Analysis of the startle response elicited by the 120 dB pulse revealed no effect of genotype (*F*_1,54_ = 2.40, *P* > .05), sex (*F*_1,54_ = 1.29, *P* > .05), or genotype × sex interaction (*F*_1,54_ = 0.24, *P* > .05; supplementary table 1A). Repeated measures ANOVA revealed habituation of the startle response (*F*_1,54_ = 14.80, *P* < .01). No significant effect of genotype (*F*_1,54_ = 3.06, *P* > .05), sex (*F*_1,54_ = 1.62, *P* > .05), or genotype × sex (*F*_1,54_ = 0.05, *P* > .05) was shown in relation to startle habituation (supplementary figure 1).

#### Prepulse Inhibition.

A repeated measures ANOVA showed that there was a significant effect of prepulse intensity on % PPI (*F*_3,162_ = 4.44, *P* < .01; figure 1C). *Nrg1* mutants demonstrated significant disruption in PPI levels (*F*_1,54_ = 5.72, *P* < .05), but this genotypic effect was not modulated by pre-pulse intensity level (*F*_1,54_ = 1.65, *P* > .05; figure 1C). No effect of sex (*F*_1,54_ = 0.001, *P* > .05), or genotype × sex interaction (*F*_1,54_ = 0.25, *P* > .05) was observed.

### Effects of Acute First- and Second-Generation Antipsychotic Drug Administration on PPI in TM-Domain Nrg1 Mutants

These PPI data were collected at the Royal College of Surgeons in Ireland (RCSI), Dublin, Ireland (see Methods and online supplementary material for study design and methodology).

#### Startle Response/Habituation.

No significant effect of *Nrg1* genotype, treatment or genotype × treatment interaction was observed in relation to startle response to the 100 dB (genotype, *F*_1,61_ = 2.53, *P* > .05; treatment, *F*_3,61_ = 1.64, *P* > .05; genotype × treatment, *F*_3,61_ = 0.37, *P* > .05), 110 dB (genotype, *F*_1,61_ = 0.10, *P* > .05; treatment, *F*_3,61_ = 2.13, *P* > .05; genotype × treatment, *F*_3,61_ = 0.61, *P* > .05), or 120 dB pulse-alone intensities (genotype, *F*_1,61_ = 2.53, *P* > .05; treatment, *F*_3,61_ = 1.64, *P* > .05; genotype × treatment, *F*_3,61_ = 0.37, *P* > .05; supplementary table 1B). Measurement of startle responsivity before and after trial blocks demonstrated habituation at a pulse-alone intensity of 120 dB (trial block: *F*_1,61_ = 5.29, *P* < .05), but no habituation of startle response was observed at the 100 dB pulse-alone intensity (trial block: *F*_1,61_ = 0.99, *P* > .05) or the 110 dB pulse-alone intensity (trial block: *F*_1,61_ = 2.41, *P* > .05). At the 100 dB pulse-alone intensity, antipsychotic treatment impacted upon startle habituation (treatment × trial block interaction: *F*_3,61_ = 4.64, *P* < .01); individual vehicle vs treatment ANOVA comparisons revealed that habituation of startle response was observed in amisulpride-treated mice only (*F*_1,36_ = 11.19, *P* < .01). Similarly, at the 120 dB pulse-alone intensity, antipsychotic treatment significantly altered startle habituation (treatment × trial interaction: *F*_3,61_ = 3.82, *P* < .05). Again, individual vehicle vs treatment ANOVA comparisons demonstrated that startle habituation was shown in amisulpride-treated mice only (*F*_1,36_ = 11.19, *P* < .01; supplementary figure 2).

#### Prepulse Inhibition.

**Fig. 2.**
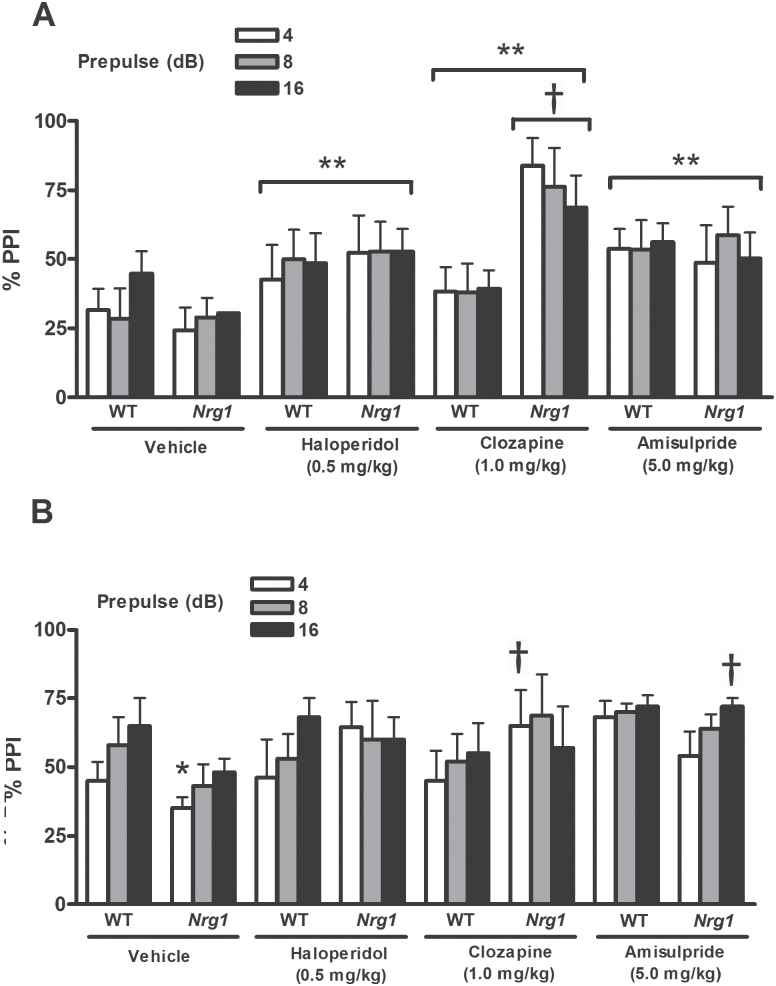
Effects of acute antipsychotic administration on pre-pulse inhibition (PPI) disruption in *Nrg1* mutants. (A) PPI in *Nrg1* mutants vs WT is selectively enhanced by acute administration of clozapine (1.0 mg/kg) at the 100 dB pulse intensity level across all of the prepulse intensity levels (4, 8, and 16 dB above background) (*P* < .01); ^†^*P* < .01 vs vehicle-treated *Nrg1* mice. However, antipsychotic treatment also increased PPI values in a genotype-independent manner (*P* < .01); **P < .01 vs vehicle-treated controls. Data are mean % PPI ± SEM. (B) PPI disruption in vehicle-treated *Nrg1* mutants vs WT is reversed by acute administration of clozapine (1.0 mg/kg), but not haloperidol (0.5 mg/kg) or amisulpride (5.0 mg/kg) at the 120 dB pulse intensity level and 4 dB prepulse intensity (*P* < .05); *P < .05 vs vehicle-treated WT mice; ^†^*P* < .05 vs vehicle-treated *Nrg1* mutants. Amisulpride (5.0 mg/kg) increased PPI at the 120 dB pulse intensity and 16 dB prepulse intensity levels in *Nrg1* mutants (*P* < .05); ^†^*P* < .05 vs vehicle-treated *Nrg1* mutants). These data are mean % PPI ± SEM. Data were collected at Royal College of Surgeons in Ireland (RCSI), Dublin, Ireland (see online supplementary material).

At the 100 dB pulse-alone intensity, antipsychotic treatment enhanced PPI in a genotype-independent manner (treatment, *F*_2,61_ = 5.03, *P* < .01; vehicle vs amisulpride, *F*_1,38_ = 9.61, *P* < .01; vehicle vs haloperidol, *F*_1,36_ = 5.83, *P* < .05; vehicle vs clozapine, *F*_1,39_ = 14.60, *P* < .01). However, at the same pulse intensity, clozapine exerted greater PPI enhancing effects in *Nrg1* mutants relative to other treatment conditions (genotype × treatment interaction, *F*_3,61_ = 3.51, *P* < .05; figure 2A). At the 110 dB pulse-alone intensity, no effect of genotype, sex, genotype × sex, or treatment × sex interaction was demonstrated (all *P* > .05) However, At 120 dB, *Nrg1* mutants showed disruption of PPI at the 4 dB pre-pulse condition (genotype × pre-pulse intensity interaction, *F*_2,122_ = 3.41, *P* < .05). This deficit was selectively reversed by antipsychotic treatment (genotype × pre-pulse intensity × treatment interaction, *F*_6,122_ = 3.18, *P* < .05); ANOVA comparisons for each individual treatment vs vehicle at the 4 dB prepulse condition revealed that reversal effects were restricted to mice treated with clozapine (genotype × prepulse intensity × treatment interaction, *F*_2,74_ = 4.98, *P* < 0.05; figure 2B). At the 16 dB prepulse condition, antipsychotic treatment selectively increased PPI at the 16 dB pre-pulse condition in *Nrg1* mutants (genotype × prepulse intensity × treatment interaction, *F*_6,122_ = 3.18, *P* < .05); individual ANOVA comparisons revealed that this treatment vs vehicle difference was observed only in mice administered amisulpride (genotype × prepulse intensity × treatment interaction, *F*_2,72_ = 3.82, *P* < .05; figure 2B). At 120 dB, haloperidol administration did not significantly alter PPI in either *Nrg1* or WT mice (treatment, *F*_1,34_ = 0.77, *P* > .05; genotype × treatment interaction, *F*_1,34_ = 1.29, *P* > .05).

### *Episodic Memory in TM-Domain* Nrg1 *Mutants*

#### Recency Discrimination [“What–When”] Analysis Using Mean Discrimination Ratios.

**Fig. 3.**
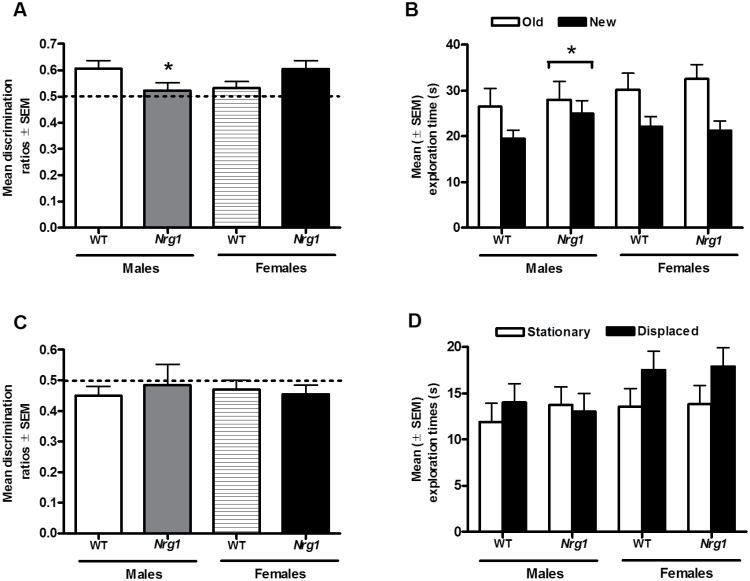
Episodic memory in Nrg1 HET vs WT mice using the “*what–where–when*” task. (A) Based on the discrimination ratio measure, male *Nrg1* mutants display increased preference for “old” vs “new” objects relative to male WT, and this effect is contrary to the preference observed for female *Nrg1* mutants and WT (*P* < .05); *P < .05 vs WT mice. Data are mean discrimination ratios ± SEM. Dashed line = chance level (50%). (B) Increased time (sec) spent exploring recently introduced objects (“new”) vs old/familiar (“old”) objects (*P* < .05) during the test for recency-based discrimination. Split by sex, male *Nrg1* mutants demonstrated significantly more time exploring the “old” object relative to the “new” object (*P* < .05); *P < .05 vs WT mice. No effect of genotype on preference for exploring “old” vs “new” was observed for female mice. Data are mean total exploration time (sec) ± SEM. (C) Using discrimination ratios, no significant difference in the exploration of “stationary” vs “displaced” objects was observed in either *Nrg1* mutants or WT. Data are mean discrimination ratios ± SEM. Dashed line = chance level (50%). (D) No significant difference in the time spent engaged in exploration of “stationary” or “displaced” objects was seen for either *Nrg1* mutants or WT mice (*P* > .05). No effect of genotype or sex. Data are mean total exploration time (sec) ± SEM.

One-way between-groups ANOVA on preference for old vs new objects as a discrimination ratio demonstrated a genotype × sex interaction (*F*_1,27_ = 4.80, *P* < .05). On splitting the data by sex, while female *Nrg1* mutants did not differ from WT in preference for old vs new objects (*F*_1,14_ = 1.42, *P* > .05), male *Nrg1* mutants showed reduced preference for old vs new objects relative to WT (*F*_1,13_ = 5.745, *P* < .05; figure 3A).

#### Recency Discrimination [“What–When”] Analysis Using Mean Exploration Times.

Repeated measures ANOVA showed a main effect of familiarity in terms of mean time spent exploring old (previously introduced) objects vs new (recently introduced) objects (*F*_1,27_ = 28.91, *P* < .001). Mean time spent exploring old objects vs new objects was unaffected by genotype (*F*_1,27_ = 0.65, *P* > .05), sex (*F*_1,27_ = 2.17, *P* > .05), and the genotype × sex interaction did not reach statistical significance (*F*_1,27_ = 2.31, *P* = .10). To further explore putative sex-specific effects of TM-domain *Nrg1* mutation,^34^ repeated measures ANOVAs split by sex were conducted. In females, while familiarity influenced mean time spent exploring old vs new objects (*F*_1,14_ = 16.14, *P* < .01), no effect of genotype was evident (*F*_1,14_ = 0.39, *P* > .05). In males, while familiarity influenced mean time spent exploring old vs new objects (*F*_1,14_ = 13.56, *P* < .01), *Nrg1* mutants demonstrated reduced time exploring new objects, reflecting short-term memory impairment in this “what–when” domain (*F*_1,14_ = 5.73, *P* < .05; figure 3B).

#### Recency Discrimination [“What–Where”] Analysis Using Discrimination Ratios.

One-way between-groups ANOVA on preference for stationary vs displaced objects as a discrimination ratio showed no effect of genotype (*F*_1,27_ = 0.14, *P* > .05), sex (*F*_1,27_ = 0.09, *P* > .05), or genotype × sex interaction (*F*_1,27_ = 0.22, *P* > .05; figure 3C). One sample *t*-tests indicated that neither WT (*t*_15_ = 1.26, *P* > .05) nor *Nrg1* (*t*_15_ = 0.68, *P* > .05) animals showed an increased preference for the displaced vs stationary object.

#### Recency Discrimination [“What–Where”] Analysis Using Exploration Times.

Repeated measures ANOVA showed no main effect of displacement on mean time spent exploring stationary objects vs displaced objects (*F*_1,27_ = 2.17, *P* > .05). There were no effects of genotype (*F*_1,27_ = 0.21, *P* > .05), sex (*F*_1,27_ = 1.03, *P* > .05), or genotype × sex interaction (*F*_1,27_ = 0.25, *P* > .05; figure 3D).

### *Comparative Metabolomic Analysis of Brain Tissue in TM-Domain* Nrg1 *Mutants*

**Fig. 4.**
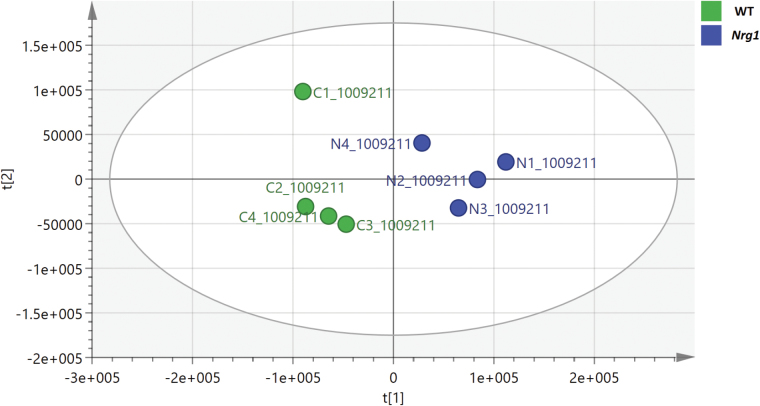
PCA separation of *Nrg1* (*n* = 4) and WT (*n* = 4) mice based on 277 metabolites in positive ion mode.

To determine the in vivo actions of TM-domain mutation of *Nrg1*, we applied high-resolution mass spectrometry to *Nrg1* mutants and WT littermate brain tissue. From the results of PCA, it was observed that genotype had a significant influence on the metabolite profiles observed because both groups (*Nrg1* and WT) were clearly distinguishable (figure 4). Significant changes in multiple polar metabolites and associated pathways were observed (tables 1 and 2). Of particular note was the evidence for aberrant lipid metabolism, particularly down-regulation of phosphatidylcholine (PC) and phosphatidylethanolamine (PE) lipid classes, in the *Nrg1* mutant. In addition, abnormalities in multiple brain metabolites across both purine metabolism and neuraminic pathways in *Nrg1* mutants provide convergent evidence for an influence of NRG1 on these processes. Increased levels of serine, 4-aminobutanoate, and acetylcholine are consistent with the known involvement of *NRG1* in neurotransmitter function.^13^

**Table 1.** Identified Lipid Metabolites Following High-Resolution Mass Spectrometric-Based Metabolomic Analysis of Male *Nrg1* Mutant Vs WT Brain Tissue

Name	MW	Rt	Ratio	*P* Value	Direction
PI38:4	887.5644	4.3	0.49	.00979	↓
PI38:5	885.5483	4.3	0.33	.00222	↓
PS 44:12	880.5121	4.7	0.39	.01298	↓
PI 36:4	859.533	4.3	0.41	.00376	↓
PE44:10	840.5519	4.9	0.41	.04708	↓
PC40:4	838.6319	5.5	0.30	.00316	↓
PI34:1	837.547	4.7	0.51	.00266	↓
PS40:6	836.5434	4.7	0.51	.00296	↓
PS 40:7	834.5277	4.7	0.35	.00204	↓
PC40:7	832.5847	5.4	0.30	.02157	↓
PC38:5	808.5843	5.5	0.31	.00468	↓
PC38:5	808.5841	6.7	0.41	.00018	↓
3-O-Sulfogalactosylceramide C18:0	808.5601	4.1	0.58	.02621	↓
PC38:6	806.5694	6.7	0.44	.00320	↓
PE40:4	796.5853	5.0	0.50	.03287	↓
Acyl phosphatidylglycerol (n-C12:0)	793.5567	5.0	0.49	.00200	↓
PE40:6	792.5532	4.9	0.47	.00591	↓
PE40:7	790.5374	5.0	0.44	.00209	↓
PC36:1	788.616	5.5	0.45	.00495	↓
PC36:2	786.6006	5.5	0.41	.00739	↓
PC36:4	782.569	6.7	0.39	.00150	↓
PE40:2 ether lipid	780.5902	4.9	0.47	.00431	↓
PG36:0	779.5781	4.9	0.46	.01456	↓
PE40:5 ether lipid	778.573	4.9	0.45	.04059	↓
PG36:1	777.562	4.9	0.48	.00113	↓
PG36:2	775.5463	4.9	0.42	.00052	↓
PE40:7 ether lipid	776.5585	4.9	0.45	.00286	↓
PE40:7 ether lipid	774.5428	4.9	0.43	.00252	↓
PE38:4	768.5534	5.0	0.47	.00034	↓
PE38:5	766.5378	5.0	0.48	.00748	↓
PE38:6	764.5221	5.0	0.48	.00125	↓
PC34:0	762.6007	6.7	0.44	.00121	↓
PC34:1	760.5846	5.5	0.45	.00276	↓
PC34:2	758.5694	6.7	0.47	.00220	↓
PE38:5	752.5586	5.0	0.44	.00910	↓
PG34:0	751.5465	5.0	0.44	.00329	↓
PE38:5 ether lipid	750.5431	5.0	0.45	.00756	↓
PG34:1	749.5308	5.0	0.48	.00018	↓
PE38:7	748.5272	5.0	0.46	.00015	↓
PC 34:0 ether lipid	746.6054	5.5	0.39	.02468	↓
PE36:2	744.5535	5.0	0.53	.02876	↓
PE36:4	740.5223	5.0	0.47	.00056	↓
PC32:0	734.5691	5.5	0.48	.00285	↓
PC32:1	732.5543	5.5	0.37	.00094	↓
SM36:2	731.6058	6.8	0.42	.00186	↓
SM36:2	729.5898	6.8	0.43	.00599	↓
PE36:3 ether lipid	726.5433	5.0	0.39	.02084	↓
PE ether lipid 36:1	724.5273	5.0	0.49	.00039	↓
PC32:2	720.5899	6.8	0.39	.00308	↓
PE34:1	718.538	5.0	0.52	.00339	↓
PC30:0	706.5379	5.5	0.47	.00348	↓
SPPC 16:0	703.5743	6.8	0.44	.00646	↓
PE34:1 ether lipid	702.543	5.0	0.56	.04100	↓
SP 18:0	566.551	4.7	0.49	.00766	↓
SP 18:1	564.535	4.7	0.64	.03157	↓
LPC18:0	524.3713	5.6	0.67	.01522	↓
Dehydrosphinganine	300.2898	9.7	0.34	.01826	↓
Choline phosphate	184.0734	20.1	1.89	.01232	↑
Choline	104.1071	17.6	1.72	.03941	↑

*Note:* Assignment of metabolites, molecular weights (MW), retention times (Rt), ratio values, *P* values, and direction of difference for *Nrg1* mutants vs WT. All metabolites with *P* < .05 were significant after application of false discovery rate (FDR) statistics to the 287 metabolites observed in positive ion mode. Codes for lipids: PI, phosphoinositol; PS, phosphoserine; PE, phosphoethanolamine; PC, phosphocholine; PG, phosphoglycerol; SM, sphingomyelin; SP, sphingosine.

**Table 2.** Identified Metabolites and Metabolic Pathways Following High-Resolution Mass Spectrometric-Based Metabolomic Analysis of Male *Nrg1* Mutant Vs WT Brain Tissue

Category + Name	MW	Rt	Ratio	*P* Value	Direction
Neurotransmitters					
Acetylcholine	146.1176	15.5	2.84	.00122	↑
Serine	106.0500	17.1	1.37	.02371	↑
4-Aminobutanoate	104.0707	17.8	1.22	.04131	↑
Amino acids					
Alanine	90.05508	16.6	1.19	.02528	↑
Leucine	132.102	13.5	3.43	.01247	↑
Asparagine	133.0609	16.9	1.92	.01548	↑
Histidine	156.0768	37.4	0.13	.00116	↓
Amino sugar metabolism					
Glycosamine acetate	222.0972	10.9	1.29	.04619	↑
N-acetylneuraminate	310.1133	12.4	1.23	.04832	↑
Muramic acid	252.1081	16.1	1.23	.00223	↑
Purines and pyrimidines					
Guanine	152.0566	15.8	0.20	.03994	↓
Cytidine	244.0929	18.9	1.35	.03496	↑
Uridine	245.0768	8.3	1.29	.03911	↑
dIMP	333.06	15.5	2.67	.00882	↑
Miscellaneous					
Orthophosphate	98.98429	15.1	1.62	.02165	↑
Nicotinamide	123.0554	9.9	1.25	.03196	↑
DL-glyceraldehyde 3-phosphate	171.0053	14.0	4.81	.04883	↑
Phosphocreatine	212.0432	13.7	0.14	.04271	↓
Glutathione	308.0911	14.0	1.36	.03052	↑

*Note:* Assignment of metabolites, classification of metabolic pathways, molecular weights (MW), retention times (Rt), ratio values, *P* values, and direction of difference for *Nrg1* mutants vs WT. All metabolites with *P* < .05 were significant after application of false discovery rate (FDR) statistics to the 287 metabolites observed in positive ion mode.

## Discussion

LI is a paradigm with high construct validity to model the disrupted attentional salience processes of schizophrenia, and has received robust validation as a pharmacological and genetic model of processes related to schizophrenia.^20^ The majority of studies have identified abnormal LI, as either inappropriately present or absent depending on symptom profile, in schizophrenia patients, patients’ relatives and individuals with high, psychometrically defined levels of schizotypy.^25^ Previous studies conducted in mutant mice containing heterozygous deletion of the *Nrg1* Type 1 immunoglobulin-like (Ig) domain demonstrated a selective LI deficit in terms of an ambulatory activity-based LI measure.^46^ However, it should be noted that the latter study did not utilize appropriate non–pre-exposed condition controls, thereby rendering the result somewhat inconclusive. In addition, on a background of reports of up-regulation of NRG1 protein and increased NRG1 signaling in schizophrenia patients,^4^ neonatal, peripheral administration of NRG1 type 1 protein in mice has been shown to produce adult deficits in PPI and LI using a fear conditioning task, which were reversed by antipsychotic treatment.^47^ The present study demonstrated intact LI in *Nrg1* mutants, with male *Nrg1* mutants exhibiting a nonspecific decrease in suppression ratios across both the pre-exposed and non–pre-exposed conditions. Reconciling contradictory data concerning the association between *NRG1* and LI performance involves acceptance of differing underlying neural mechanisms governing the effects of mutation of *Nrg1* on attentional tasks of relevance to schizophrenia. NRG1/ErbB4 activation modulates the signaling of cortical GABA interneurons,^48^ and is also involved in glutamatergic^49^ and dopaminergic transmission.^45,50,51^ It has been proposed that the frontal-cingulate cortical circuit is implicated in NRG1-mediated function of attentional salience.^48^

Reduced PPI has been reported in humans carrying the rs3924999 mutation of the *NRG1* gene, and patients with schizophrenia who exhibit abnormal PPI over-express this mutation relative to controls.^52^ Isoform-specific *Nrg1* or *ErbB4* receptor gene hypomorphic mice, as well as mice genetically over-expressing the *Nrg1* type I isoform, show disruption of PPI across a variety of parametric conditions.^46,53,54^ In contrast, phenotypic studies involving the TM-domain *Nrg1* mutant have suggested that PPI deficits may not be a robust effect in this line; rather, PPI deficits in TM-domain *Nrg1* mutants are suggested to be protocol- and site-specific.^18,30^ In the present study, PPI was measured in TM-domain *Nrg1* mutants housed in separate laboratories in Dublin and Nottingham, as well as using different testing protocols. PPI disruption in *Nrg1* mutants was consistently observed across both sites and testing conditions, which is consistent with some of the existing literature.^43,55,56^ Karl and colleagues^57^ have previously demonstrated environmental modification of expression of psychosis-relevant phenotypes in this *Nrg1* mutant line, and have reported particular sensitivity of the PPI phenotype of the *Nrg1* mutant to housing conditions and test protocols when tested in several Australian laboratories.^18^ Their conclusions are in line with the present study findings, and may also provide insight into discrepant *Nrg1* × PPI findings between Australia, Dublin, and Nottingham. Although PPI disruption was demonstrated in *Nrg1* mice in both the Dublin and Nottingham test facilities, the nature and magnitude of the PPI deficit differed across both sites; other reported PPI findings conducted in Dublin-based *Nrg1* mutants have shown similar phenotypic variability.^55,56^ In the original report of PPI deficits in the TM-domain *Nrg1* mutant,^43^ employing a similar protocol, mutants displayed a 10%–15% reduction in PPI relative to controls. In other studies where protocol-specific modification of PPI has been observed, genotypic effects have been restricted to specific prepulse intensities, and a similarly modest deviation from the WT profile has been reported.^18^ Behavioral phenotypes including increased novelty-induced hyperactivity and social interaction deficits, have been reliably and robustly observed in the TM-domain *Nrg1* mutant.^34^ In contrast, both the Dublin and Nottingham PPI results indicate a very mild PPI deficit in this *Nrg1* mutant line; data from our laboratory and others have confirmed that this *Nrg1*-related PPI deficit is markedly sensitive to acute and, in particular, prolonged exposure to stress, as well as test protocols and minor environmental modifications.^37,55^

Antipsychotic treatment is associated with differential *NRG1/ErbB4* expression in both brain and serum in human and animal studies.^58–60^ In patients with first-episode schizophrenia, serum NRG1 expression was increased following 2 weeks of treatment with risperidone or quetiapine.^59^ NRG1-ErbB4 function is also involved in several neurotransmitter pathways implicated in antipsychotic activity.^59^ Treatment with clozapine (where clinical efficacy is associated with antagonism at 5-HT2A, 5-HT2C, and other receptors in addition to dopamine D2-like receptors) has been shown to reverse both PPI deficits in *ErbB4* partial knockout mice^60^ and LI in Ig-like domain *Nrg1* mutant mice.^46^ However, while clozapine selectively reversed novelty-induced hyperactivity in TM-domain *Nrg1* mutants, an initial report suggested that it may not be effective against PPI disruption in this line.^43^ In the current study, it was demonstrated that clozapine partially restored disrupted PPI in *Nrg1* mutants at the 120dB pulse intensity, without altering baseline startle reactivity or PPI in WT. At the lower pulse intensity of 100 dB, clozapine treatment selectively enhanced PPI in *Nrg1* mutants, but a generalized increase in PPI values under these conditions was observed across all treatment conditions relative to vehicle-treated controls.

Haloperidol (a dopamine D2-like receptor antagonist) displayed no effect on *Nrg1*-mediated PPI disruption, while amisulpride (a dopamine D2/D3 receptor antagonist) selectively increased PPI in *Nrg1* mutants at the 120 dB pulse intensity, at a prepulse intensity (16 dB) which was intact in vehicle-treated *Nrg1* mutants vs WT controls. In light of the pleiotropic role for NRG1 in neuronal function, the locus of interaction between NRG1/ErbB4 and antipsychotic activity is unclear. However, decreased phosphorylation of the NR2B subunit in brains of TM-domain *Nrg1* mutants is normalized by clozapine,^63^ suggesting a glutamatergic basis for this interaction. The present PPI results confirm a role for the NRG1-ErbB4 signaling pathway as a putative modulator of antipsychotic drug effects in this paradigm, as well as a target for discovery of new antipsychotic drugs.^64,65^

**Fig. 5.**
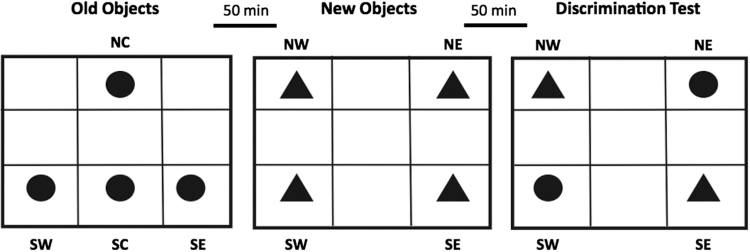
Schematic drawing of the episodic memory task encompassing the “what,” “where,” and “when” components. Object locations: NC, north-center; SW, south-west; SC, south-center; SE, south-east; NW, north-west; NE, north-east. Adapted from Dere et al.^67^

fMRI analyses conducted in healthy subjects have demonstrated that variation in the single nucleotide polymorphism rs35753505 in the *NRG1* gene is associated with differences in activation of specific brain areas during completion of an episodic memory task.^66^ Specifically, activation in the cingulate gyrus, left middle frontal gyrus, bilateral fusiform gyrus, and left middle occipital gyrus was modulated by *NRG1* genotype during the *encoding* phase of the task. During the *retrieval* phase, left middle occipital gyrus activation was associated with *NRG1* variation. However, this study failed to demonstrate any effect of *NRG1* genotype on task performance. Studies employing the TM-domain *Nrg1* mutant have demonstrated intact spatial learning and working memory,^34^ but impaired novel object recognition memory.^35^ We sought to investigate the possibility of phenotypic effects of *Nrg1* mutation on the recency-mediated “*what–when*” and “*what–where*” object recognition components of an episodic memory task (figure 5). The results indicated that male *Nrg1* mutants show impairment in their memory for the “*what–when*” component of the episodic memory task, which was marked by increased exploration for an old vs new object.

No conclusions could be drawn with respect to the spatio-temporal (“*what–where*”) component of this task, as neither WT nor *Nrg1* mutants showed a preference for exploring the stationary vs displaced objects. The absence of exploratory preference on the “*what–where*” component may be interpreted as an absence of spatial learning in both WT and *Nrg1* mutants.^67^ However, it has been proposed that the absence of any preference in object exploration does not necessarily reflect a learning deficit. Episodic memory tested in a single day may be influenced by delays (50 min in the present study) between sample and test phases; at short delays, it is suggested that memory for a familiar object is intact and at longer delays it becomes weak.^68^ The absence of preference in the spatial aspect of the task may be attributed to a decline in interest for object exploration in this third phase (sample 1, sample 2, and test) of object exposure; rates of exploration for “*what–where*” (stationary vs displaced) objects are greatly reduced compared to exploration rates for “*what–when*” (old vs new) objects. Alternatively, spatial discrimination memory may be subject to interaction with recency.^69^ A recent study confirmed that the episodic memory task employed in the present study is subject to paradigmatic influences and indicates that the spatio-temporal interaction in the existing protocol may confound recency-mediated memories.^67^ It should be noted, however, that the temporal element cannot be removed from the spatial memory aspect of this task, and this is not a standalone measure of spatial memory, but, rather, of spatio-temporal memory. In summary, these findings demonstrate that reduced function in the TM domain of *Nrg1* has sex-specific effects on episodic-like memory via impairment of recency-based novel object discrimination in male *Nrg1* mutants.

Lipids and constituent fatty acids are crucial for diverse functional roles in the brain, including membrane composition and signal transduction, which are compromised in schizophrenia.^70,71^ Studies employing dedicated lipidomics platforms have reported significant changes in phosphotidylcholine (PC) and phosphotidylethanolamine (PE) groups, both of which are implicated in membrane composition, in schizophrenia patients, in a manner unrelated to antipsychotic treatment.^72,73^ More recent studies have demonstrated structural lipid alterations in brain areas implicated in schizophrenia in patients as well as mice mutant for a schizophrenia risk gene (*G72/G30*).^74^ It has been suggested that these modifications in lipid metabolic pathways may contribute to neural dysconnectivity in schizophrenia.^73^ Consistent with our findings in the *Nrg1* mutant, significantly lower concentrations of PC have been detected in postmortem brain tissue of patients with schizophrenia.^76,77^ The present study reported a specific decrease in PC 38.6 in *Nrg1* mutant brain; postmortem brain analyses, as well as studies conducted in plasma and platelet plasmalogens of patients with schizophrenia, have revealed similarly decreased levels of PC 38:6 in schizophrenia.^76,78^ PC 38:6 and its associated interacting proteins has been shown to play a role in diverse immune-related and neurotrophin signaling pathways^79^; the latter pathway, in particular, plays an important role in the regulation of neuron differentiation and proliferation during brain development, as well as participating in neuronal plasticity process associated with learning and memory.^80^ As NRG1 is a neurotrophic factor, these analyses are suggestive of mechanisms by which genetic factors associated with schizophrenia may modify signaling pathways linked with disturbance of cognition in schizophrenia.

TM-domain *Nrg1* mutant mice demonstrate differential susceptibility to several of the neurobehavioral and cellular/molecular effects of acute and chronic tetrahydrocannabinol (THC) and other cannabinoids relative to wildtype controls.^81–83^ A recent study demonstrated abnormal concentrations of two lipids in the brain of TM-domain *Nrg1* mice, increased endogenous cannabinoid anandamide in the amygdala and decreased 2-arachidonoylglycerol (2-AG) in the hypothalamus.^84^ In animal tissue, anandamide is generated from its membrane precursor the glycerophospholipid N-arachidonoyl phosphatidylethanolamine (NAPE). The present finding of abnormal glycerophospholipids such as choline in TM-domain *Nrg1* mutant mice, taken together with the evidence that the endocannabinoid system is dysregulated in schizophrenia,^85^ highlight the importance of further research to advance our understanding of the mechanisms underlying the interaction between NRG1, the endocannabinoid system, and schizophrenia.

Several metabolites belonging to the neuraminate pathway were significantly affected in *Nrg1* mutants. The most marked accumulation is of N-acetylneuraminic acid, the most abundant form of sialic acid found in mammalian cells. Sialic acid in the form of polysialic acid has been reported to be involved in schizophrenia and schizophrenia-like symptoms.^86–88^ Previous examination of differentially expressed proteins in the hippocampi of transmembrane-domain *Nrg1* vs WT controls identified significantly decreased expression of fibroblast growth factor 14 (FGF14) and other growth factors.^83^ Polysialic acid, a linear polymer of sialic acid, has been shown to specifically bind fibroblast growth factors,^89^ and recent work has demonstrated that polysialic acid also exerts protective effects of on proteolytic cleavage of FGF2.^90^ Elevated fibroblast growth factor receptor mRNA has been reported in the prefrontal cortex of patients with schizophrenia relative to controls.^91^ In addition, mice with genetic knockout of *FGF14* demonstrate cognitive deficits analogous to those observed in schizophrenia, together with altered expression of cellular and molecular components of GABA and glutamatergic function in schizophrenia-associated brain areas.^92^ Further studies of the relationship between sialic acid and growth factors including NRG1 and FGFs are clearly warranted.

*Nrg1* mutants also demonstrated altered brain expression of metabolites associated with the purine metabolism pathway. Homeostatic imbalance of purine catabolism has previously been reported in first-episode, antipsychotic-naive patients with schizophrenia.^93^ Allopurinol, which acts as an inhibitor for xanthine oxidase, an enzyme involved in purine metabolism, has been proposed as an adjunctive therapy for schizophrenia.^94^ It has been suggested that purine metabolism is an important contributor to the pathophysiological process that links oxidative stress to membrane dysfunction, changes in key neurotransmitters, and the development of schizophrenia and other neuropsychiatric disorders.^93,95^ A significant increase of glutathione was also observed in *Nrg1* mutants relative to WT. Glutathione alleviates the effects of oxidative stress, acting as an antioxidant protecting cells from damage induced by reactive oxygen species, and altered glutathione metabolism has been reported in patients with schizophrenia.^96^

These results demonstrate that TM-domain *Nrg1* mutant mice demonstrate specific cognitive and metabolic phenotypes related to schizophrenia, including a mild PPI impairment which is sensitive to antipsychotic treatment, as well as a sexually dimorphic deficit in the “*what–when*” element of an episodic memory task. *Nrg1* mutants also displayed alterations in lipid metabolism and other metabolic pathways implicated in pathophysiological processes associated with schizophrenia. Several disorders, including metabolic syndrome, diabetes, and cardiovascular disease, are characterized by changes in lipid metabolism. Patients with schizophrenia are at increased risk for metabolic abnormalities,^96,97^ and several antipsychotic drugs, particularly clozapine and olanzapine, can cause adverse effects that are related to lipid metabolism (eg, weight gain, insulin resistance).^44,98^ This study has identified novel physiological mechanisms by which disruption of NRG1 function might contribute to the emergence of schizophrenia-relevant phenotypes in adulthood.

## Supplementary Material

Supplementary data are found at *Schizophrenia Bulletin* online.

## Funding

This work was supported by the Wellcome Trust [grant number WT0845921Z], UK; and Science Foundation Ireland (07 / IN.1 / B960).

## Supplementary Material

Supplement Materials
