# Supplementary material for: Specialized Information Processing Deficits and Distinct Metabolomic Profiles Following TM-Domain Disruption of Nrg1
Source: Schizophr Bull. 2017 Mar 11;43(5):1100–13. doi: 10.1093/schbul/sbw189 (PMC5581893; doi:10.1093/schbul/sbw189)
Supplement: Supplement Materials [file sbw189_suppl_Supplement_Materials.doc]

**SUPPLEMENTARY MATERIAL**

**Specialised information processing deficits and distinct metabolomic profiles following TM-domain disruption of *Nrg1***

O’Tuathaigh CMP, Mathur N, O'Callaghan MJ, MacIntyre L, Harvey R, Lai D, Waddington JL, Pickard BS, Watson DG, Moran PM

**METHODS**

**Drugs**

Clozapine (Sigma Aldrich, USA), haloperidol (Sigma Aldrich, USA), and amisulpride (Tocris Bioscience, UK) were dissolved in 5-10 μl of acetic acid, adjusted to 6.5 pH using NaOH, and diluted with physiological saline (0.9% NaCl). Antipsychotic drug doses were selected on the basis of studies showing relevant antipsychotic effects on psychostimulant-induced hyperactivity or PPI disruption in mice ^1, 2^. All drugs were injected subcutaneously at a volume of 4 ml/kg.

**LI**

Training and testing was conducted in six identical light- and sound-attenuating conditioning chambers (21.6 cm × 17.8 cm × 12.7 cm, ENV-307W, MED Associates Inc., VT, USA). These were composed of Plexiglas walls at the front and back of the chambers, and two stainless steel sides. In addition, a metal floor grid was connected to a shock generator. Each box contained a ventilation fan, mounted above the chambers to provide an inflow of air, and background noise (69 dB white noise). A Sonalert module (ENV-323AW, Med Associates Inc., VT, USA) was mounted on a wall for delivering the conditioned stimulus (CS; 85 dB). Each chamber was equipped with a removable drink spout located along the left wall, connected to a lickometer (ENV-250, Med Associates Inc., VT, USA) that recorded the number of licks made by the animal. The chambers were connected to a PC computer that employed MED-PC software (SOF-735, MED Associates Inc., VT, USA) to control stimulus presentation and record data. The LI protocol ^3^ comprised six stages: water restriction; pre-training; pre-exposure/non-pre-exposure; conditioning; re-establishment of drinking; and testing.

Water restriction [days 1–7]: Mice were placed on a 23-h water restriction schedule 7 d before pre-training began. Water in the test apparatus was given in addition to the daily ration of 1 h delivered in the home cages. This regimen was maintained throughout the experiment.

Pre-training [days 8–13]: Mice were placed in the conditioning chambers and allowed to drink freely from a water sipper for 15 min. The number of licks made and latency to lick was recorded.

Pre-exposure [day 14]: Mice were placed in the conditioning chambers without access to the water sipper. They were given 60 presentations of an 85-dB 5-s tone with a 15-s inter-stimulus interval. Non-pre-exposed (NPE) control mice were placed in the chambers for the same amount of time but received no pre-exposures to the tone.

Conditioning [day 15]: Mice were placed in the experimental chambers without access to the water sipper. After 5 min, two tone-footshock pairings were presented. Each tone (CS) was of 5-s duration and was followed by a 1-s 0.38-mA US footshock. There was a 2.5-min interval between pairings and mice remained in the chamber for 5 min following the second tone-shock presentation.

Lick training [days 16–17]: Mice were placed in the conditioning chambers for 15 min and given free access to the water sipper to re-establish licking in the chamber prior to testing. Mice that did not lick consistently were omitted from the experiment at this stage.

Testing [day 18]: Mice were placed in the conditioning chambers with access to the water sipper. The number of licks and the time to complete licks 80–90 (A) and 90–100 (B) were recorded electronically. After the first 90 licks, the tone CS was presented until the mouse reached lick 100. The standard measure of conditioned suppression was the time taken to complete licks 90–100 in the presence of the CS. A suppression ratio (SR) was calculated according to the formula A/(A+B) yielding a scale of 0–0.5. Low SR indicates good learning while high SR indicates poor learning of the association between the tone and footshock. LI is demonstrated as higher SR in the pre-exposed (PE) group compared to the NPE group. An effect of genotype on LI would manifest as a selective effect on the PE group; effects on NPE SR values represent a generalised learning impairment.

**PPI (Nottingham)**

Two startle chambers (SR-LAB, San Diego Instruments, CA, USA) were used to measure startle reactivity. Each chamber consisted of a sound-attenuated, lighted and ventilated cabinet holding a single Plexiglass chamber (5 cm inner diameter). All acoustic stimuli were produced by a high frequency loudspeaker mounted inside the chamber. In order to ensure consistent stabilimeter sensitivity, a calibration system was employed across both chambers. A high frequency loudspeaker produced continuous background noise of 65db, in addition to the acoustic stimuli. Vibrations of the plexiglass cylinder caused the whole startle response of the animal to be recorded as analogue signals that were then stored by a computer. Eighty-four readings were taken at stimulus onset and during the entire test session. Each session had a block of 6 × 120 dB pulses interspersed with 68dB, 72dB, 80dB and 90 dB pre-pulses.

Prior to testing the animals in the PPI apparatus, animals were given a one day habituation session, where mice were removed from their home cages and placed in the PPI apparatus for 25-30 minutes. They were left in the startle chamber for the entire duration, where white noise was tuned on but no tone were presented. The PPI test session consisted of a one-day session of startle trials (pulse-alone) and pre-pulse trials (pre-pulse + pulse), intermixed with no-stimulus trials. The pulse-alone trial consisted of a 40 ms, 120 dB pulses of noise. PPI was based on acoustic pre-pulse intensities that consisted of noise pre-pulses 68dB, 72dB, 80dB, and 90 dB presented for 20 ms (3, 7, 15, 25 dB above 65 dB background noise). After mice were placed in the startle chamber, a 65 dB background noise level was presented for a 10 min acclimation period and continued throughout the test session. Each session began and ended with blocks (blocks 1 and 3) of 6 pulse-alone trials that were used for measuring habituation and were not included in the analysis of PPI or mean acoustic startle response. In between these two blocks, during block 2, each trial type (pulse-alone, pre-pulse + pulse for the four pre-pulse intensities) was presented on 12 occasions in a pseudo-random order. The no-stimulus trial consisted of background noise only. There was a delay of 7 sec between trials. Two startle chambers were used for testing, and each animal was always tested in the same startle chamber throughout.

Percentage PPI (%PPI) was calculated as [(mean startle response to pulse-alone − mean startle response to pre-pulse-plus-pulse response) / mean startle response to pulse alone] × 100%; % PPI was calculated for each combination of pre-pulse / pulse-alone intensity. The acoustic startle response [ASR] to the 120 dB pulse alone trials (excluding each block of six at the beginning and end of the session) was also analysed. Habituation to the 120dB tone was calculated as: (mean initial 120dB startle - mean final 120 dB startle)/mean initial startle × 100. This habituation was a measure of baseline startle and was based on the initial block

of six 120 dB pulses at the beginning and block of six pulses at the end of the session. All scores were expressed as percentages. Pre-pulses were administered in a random order over the session.

**PPI (Dublin)**

Testing of PPI was performed in a startle chamber (SR-LAB, San Diego Instruments, CA, USA), using a procedure described previously ^4^. All animals were given a one day habituation session, where mice were removed from their home cages and placed in the PPI apparatus for 25-30 minutes. They were left in the startle chamber for the entire duration. Each test session began with a 10-min acclimatization period where the mouse was placed into the cylinder and exposed to a constant background noise of 65 dB only. Subsequently, in a pseudorandom order, mice were presented with a block of 172 discrete test trials with inter-trial intervals ranging between 5 and 15 s; each block consisted of pulse-alone trials at three different intensities (100, 110 or 120 dB); these were combined with pre-pulse signals (4, 8, or 16 dB) to give nine different combinations of pre-pulse/pulse trials (4/100, 4/110, 4/120, 8/100, 8/110, 8/120, 16/100, 16/110, 16/120 dB). The first six and last six trials consisted of startle alone, comprising two trials of each of the three possible pulse intensities. Initial and habituated startle response, at each pulse-alone intensity, was calculated on the basis of the average startle value for the first six and last six trials, respectively. Startle response was calculated as mean amplitude to all startle trials. Percentage PPI was calculated as described in the preceding section.

**‘*What-where-when*’ episodic memory task**

This task was conducted in mice as described previously (Figure 5) ^5^. Note: this task differs from many "classic" object recognition protocols in that mice have previously seen all objects there is therefore no "novel" object. Object exploration was assessed in an open-field chamber (8 boxes; 30 cm × 30 cm × 40 cm) that consisted of an enclosed box constructed from clear plastic. Two sides of each chamber were covered with black Fablon adhesive plastic, in order to prevent animals in any one chamber from seeing animals in neighbouring chambers. Multiple spatial cues were also provided, i.e. the left wall to the chamber was blue; the wall behind the chambers was covered in a white sheet with a stripe pattern; its adjacent wall displayed a large red triangle. A video camera, connected to a video recorder, was mounted 70 cm above the field to record and store activity videos on a computer for analysis. Diffuse white light provided illumination in the centre of the chamber. A fan, creating white noise, was located at the top right corner of the chamber and also acted as a cue. After each trial, the apparatus was thoroughly cleaned with a 75% ethanol solution to remove any odour cues.

*Objects***:** Two different objects (four specimens each of a glass conical bottle and a golf ball), with varying texture and made of different materials were used. The objects were cleaned thoroughly with 70 % ethanol solution so that they could not be distinguished by

odour cues. In the habituation session to a novel object, objects unrelated to those

presented in the test phase (Lego blocks) were used, so that there was no prior exposure to a similar object. Pilot studies ensured that mice could discriminate between both objects, and ascertained that there was no preference for one of these objects.

*Handling,* *Days 1-7:* Mice were habituated to the handling procedure prior to the ‘*what-where-when*’ episodic memory task for seven consecutive days. They were released into the open field for an exposure of 1 minute, after which they were returned to their home cage.

*Habituation,* *Days 8-10*: After handling, mice were familiarized to the test apparatus, while devoid of any objects, for 5 min per day over three consecutive days.

*Habituation with and without objects,* *Days 11-12*: On the following two days, to ensure habituation mice received 3 additional daily sessions with exposure for 10 minutes. Two objects (Lego blocks) were placed in the corners of the open field, and each exposure was separated by a 20 minute inter trial interval. These objects were not used in the ‘*what-where-when*’ task.

*Test of memory for ‘what-where-when’, Day 13***:** Each mouse received two sample trials and a test trial. On the first sample trial, mice were placed in the centre of the open field that contained four copies of a novel object in a triangle shaped configuration; one object was placed in the centre of the northern wall (NC), one at the south-west corner of the box (SW), one at the south–centre and one at the south-east corner of the box. Animals were allowed to explore these objects for a period of 10 minutes. After a delay of 50 minutes, animals received a second sample trial that was identical to the first, except that the four novel objects were arranged in a different spatial orientation in the open field. Objects were arranged in a quadratic formation, where one object was arranged in each of the north-west (NW), north-east (NE), south-west (SW) and south-east (SE) corners. The objects determined for each mouse were counterbalanced across both sample trials. After a second delay of 50 minutes, mice received a test trial identical to the second sample trial, where two copies of the old, familiar object were places in the SW and NE corners, in their stationary (as-before) and displaced (not as-before) positions, respectively, and two copies of the new object were placed in the NW and SE corners. The ‘*what-when’* component of the task indicates a preference for the old objects *vs* the new objects, note that this can differ from some one-trial procedures in which mice preferentially explore the novel object. The ‘*what-where’* component of the task indicates a preference for the old objects at their original (stationary) locations *vs* their subsequent (displaced) locations. Data were analysed using Discrimination ratios (DI) that expressed the preference/discrimination of one object as a proportion of the total amount of time spent exploring both objects was also calculated as follows: DI (old *vs* new ratio) = time spent at old object (in seconds)/ time spent at old object + time spent at new object; DI (stationary *vs* displaced ratio) = time spent at stationary old object/time spent at stationary old object + time spent at displaced old object. Mean exploration times in exploring the old and new objects and exploration times in exploring the stationary and displaced objects are also reported. Where we found an effect of sex on DI we also reported exploration times by sex for comparative purposes.

**Metabolomic analysis**

Metabolomic analysis for the Nrg1 mutant was conducted using whole brain samples for a numer of reasons: (a) Nrg1 is a pleiotropic growth factor involved in diverse aspects of brain development and function across different brain areas. Additionally, targets of NRG1 in neurotransmission and synaptic plasticity include GABAergic interneurons, glutamatergic neurons, and dopaminergic neurons. Hence, It was decided, initially, to conduct a more global analysis rather than focusing on specific brain areas implicated in schizophrenia and/or cognitive features of schizophrenia; (b) PPI, LI, and episodic memory (as measured in the object memory task) are dissociable in terms of mechanisms, circuits, structures, pathways etc (c) technical considerations informed the use of whole brain homogenate in the current study, based on the volume requirements for a non-targeted metabolomic assay.

For extraction of polar metabolites from brain tissue homogenates for liquid chromatography–mass spectrometry (LC–MS) analysis, brains from male *Nrg1* mutants (*n* = 4) and WT (*n* = 4) littermates were snap-frozen in isopentane and stored at -80°C. A two-step metabolite extraction method using methanol, water and chloroform as extraction solvents produced a biphasic solution comprising polar and non-polar fractions. Only the polar fractions were analyzed in this study. In all, 200 ml of collected polar extract was added to 600ml of 1:1 acetonitrile:water. Samples were diluted to combat salt interference and to prevent ion suppression. Samples were filtered using Acrodisc 13mm syringe filters with 0.2mm nylon membrane (Sigma Aldrich, UK) before LC–MS analysis.

*LC–MS analysis of polar metabolites*: Analysis was carried out using a Finnigan LTQ-Orbitrap fitted with a Surveyor HPLC pump (Thermo Fisher, Hemel Hempstead, UK) using 30 000 resolution. The software program XCalibur (version 2.0, Thermo Fisher, Hemel Hempstead, UK) was used to acquire the LC–MS data. Analyses were carried out in positive and negative modes over mass range of 60–1000 m/z. The capillary temperature was set at 250 °C and in positive ionization mode the ion spray voltage was 4.5 kV, the capillary voltage was 30 V and the tube lens voltage was 105 V. In negative ionization mode, the ion spray voltage was 4.5 kV, the capillary voltage was 25 V and the tube lens voltage was 95 V. The sheath and auxiliary gas flow rates were 45 and 15, respectively (manufacturer units). A ZIC-HILIC column (5 µm, 150 × 4.6 mm^2^; HiChrom, Reading, UK) was used with a binary gradient. Solvent A was 0.1% v/v formic acid in HPLC water and B was 0.1% formic acid in acetonitrile. A flow rate of 0.3 ml per minute was used and the injection volume was 10 ml. The gradient programme used was 80% B at 0 min to 50% B at 12 min to 20% B at 28 min to 80% B at 37 min with a total run time of 45 min. The instrument was externally calibrated before analysis and internally calibrated using lock masses at m/z 83.06037 and m/z 195.08625. Samples were analyzed sequentially and the vial tray temperature was set at a constant temperature of 4°C.

*Data Extraction and Metabolite Identification:* MZMine 2.14 was used for peak extraction and alignment, as previously described. ^6^ Putative identification of metabolites was also conducted in MZMine by searching the accurate mass against our in-house database. ^7^ Background peaks present in the blank were removed in MZmine before transferring the data to an Excel file. The GC-MS data were extracted by using Sieve 1.3 (ThermoFisher Scientific UK), and the ions corresponding to the retention time of the sugar standards were extracted in order to build the OPLS-DA model.

*Multivariate and Univariate Analysis*: All data processing, including data visualisation, biomarker identification, diagnostics, and validation was implemented using SIMCA software v.14 (Umetrics AB, Umeå, Sweden). Principal component analysis (PCA) was used to provide an unsupervised model in order to explore how variables clustered regardless Y class. ^8^ The p-values of the biomarkers were evaluated for their significance applying the false discovery rate statistic (FDR). ^9^ With an FDR procedure the greater the number of significant discoveries the more liberal the acceptance threshold.

**Data Analysis**

Statistical analysis of behavioural and psychopharmacological data was performed using SPSS software, version 20 (SPSS Inc., Chicago, IL, USA). For the LI task, independent analysis of variance (ANOVA) was used with SR as dependent variable and exposure (NPE/PE), genotype (*Nrg1* mutant/WT), and sex (male/female) as factors. For PPI conducted in Nottingham, percentage PPI was analyzed using ANOVA to compare differences in startle to pre-pulses in *Nrg1* mutants and WT, with % inhibition to each pre-pulse intensity (3dB, 7dB, 15dB, 25dB above background) as the within-subjects factor. Genotype and sex were between-groups factors. For PPI conducted in Dublin (antipsychotic treatment study), for each pulse alone intensity (100, 110, 110dB), percentage PPI was analyzed using ANOVA to compare differences in startle to pre-pulses in treated and non-treated *Nrg1* mutants and WT. % inhibition to each pre-pulse intensity (4dB, 8dB, 16dB above background) was the within-subjects factor. Genotype and antipsychotic treatment condition (vehicle, haloperidol, clozapine, and amisulpride) were the between-groups factors. Where a significant effect of treatment or genotype × treatment interaction was observed, separate ANOVA were conducted which compared each treatment condition *vs* vehicle-treated controls. For the ‘*what-where-when*’ task, data were analysed using mean exploration times and discrimination ratios, and two repeated measures ANOVAs performed with object type (old *vs* new or stationary *vs* displaced) and genotype and sex as within-groups factors. For the above phenotypic measures, we hypothesized *a priori* that the effects of mutation of *Nrg1* would be modulated by sex ^10, 11^; thus, where appropriate, separate ANOVA analyses were also conducted within male and female groups. For *post-hoc* comparisons, student's independent t tests with Bonferroni adjustment for multiple comparisons where appropriate.

**RESULTS**

**Latent Inhibition performance in TM-domain *Nrg1* mutants**

Analysis in males revealed a significant effect of exposure on SR values (F _1, 26_= 6.86, *P* < 0.05); while overall SR was decreased in NRG1Nrg1 mutants (F _1, 26_= 4.30, *P* < 0.05), no genotype by exposure interaction was evident (*P* > 0.05), confirming that LI was preserved. In females, a significant effect of exposure on SR values (F _1, 28_= 5.95, *P* < 0.05) in the absence of any other main or interaction effects (all *P* > 0.05) confirmed that LI was preserved.

**A**

| Pulse-Alone dB level | **WT** | | ***Nrg1*** | |
| --- | --- | --- | --- | --- |
|  | Males | Females | Males | Females |
| 120 | 37.6 ± 6.5 | 35.3 ± 6.0 | 35.9 ± 6.0 | 29.0 ± 5.6 |

| Pulse-Alone dB level | **WT** | | | | ***Nrg1*** | | | |
| --- | --- | --- | --- | --- | --- | --- | --- | --- |
|  | Vehicle | Haloperidol (0.5 mg/kg) | Clozapine (1.0 mg/kg) | Amisulpride (5 mg/kg) | Vehicle | Haloperidol (0.5 mg/kg) | Clozapine (1.0 mg/kg) | Amisulpride  (5 mg/kg) |
| 100 | 54.1 ± 13.3 | 23.3 ± 16.2 | 44.7 ± 15.0 | 36.5 ± 13.3 | 45.4 ± 9.9 | 39.6 ± 15.0 | 46.2 ± 13.3 | 58.3 ± 16.2 |
| 110 | 93.6 ± 20.3 | 38.5 ± 24.8 | 55.9 ± 22.9 | 66.9 ± 20.2 | 61.7 ± 15.2 | 38.4 ± 22.9 | 62.8 ± 20.2 | 89.8 ± 24.8 |
| 120 | 140.5 ± 30.3 | 51.6 ± 37.1 | 68.6 ± 34.4 | 111.2 ± 30.3 | 83.4 ± 22.7 | 61.0 ± 34.4 | 74.6 ± 30.3 | 100.5 ± 37.1 |

**B**

Supplementary Table 1: Acoustic startle response (ASR; arbitrary units) to (A) startle pulse (120 dB) in male and female WT and *Nrg1* mice based on data collected at University of Nottingham, and (B) startle pulses (100, 110, 120 dB) in WT and *Nrg1* mice across the four treatment conditions (vehicle, haloperidol (0.5 mg/kg), clozapine (1.0 mg/kg), amisulpride (5.0 mg/kg)), based on data collected at RCSI, Dublin. ASR values were averaged over the test trial. Data are presented as means ± SEM.

**
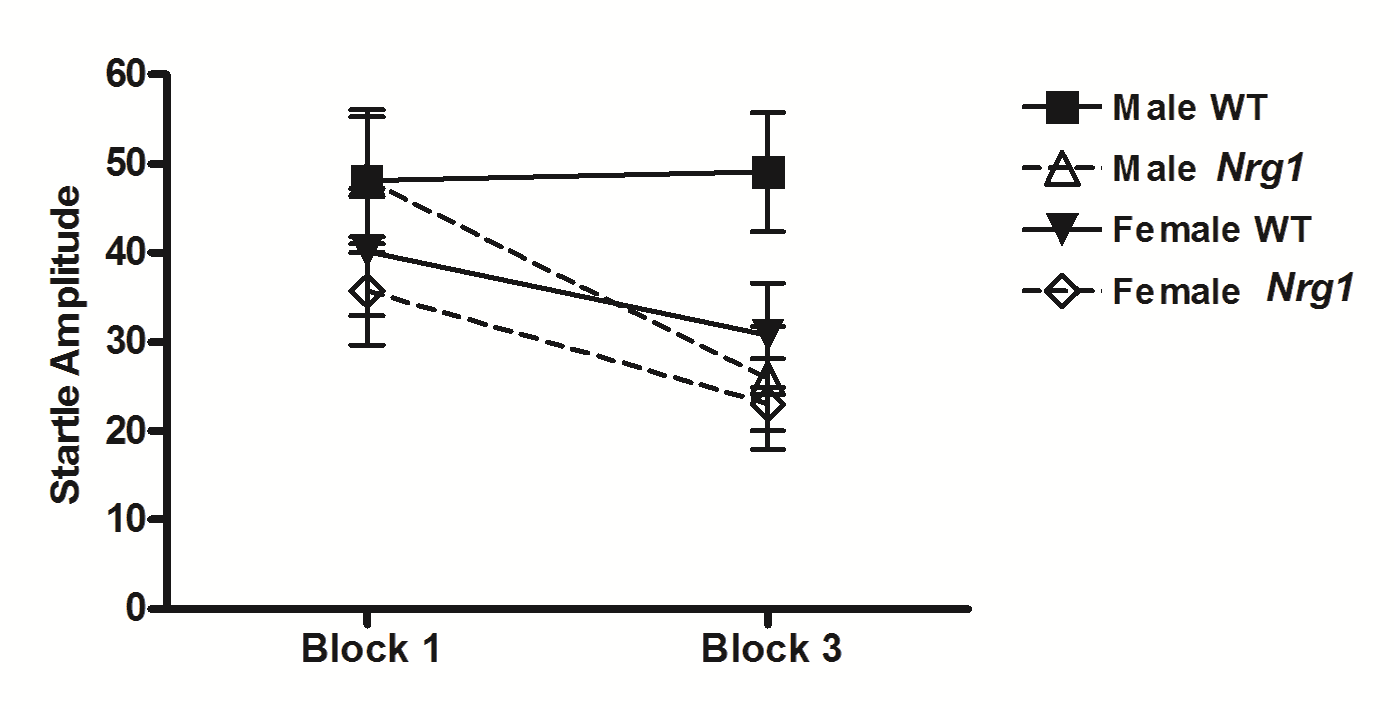
**

Supplementary Figure 1: Habituation to the acoustic startle response (ASR; arbitrary units) to a 120 dB pulse in male and female WT and *Nrg1* mice based on data collected at University of Nottingham. All data are presented as means ±SEM.


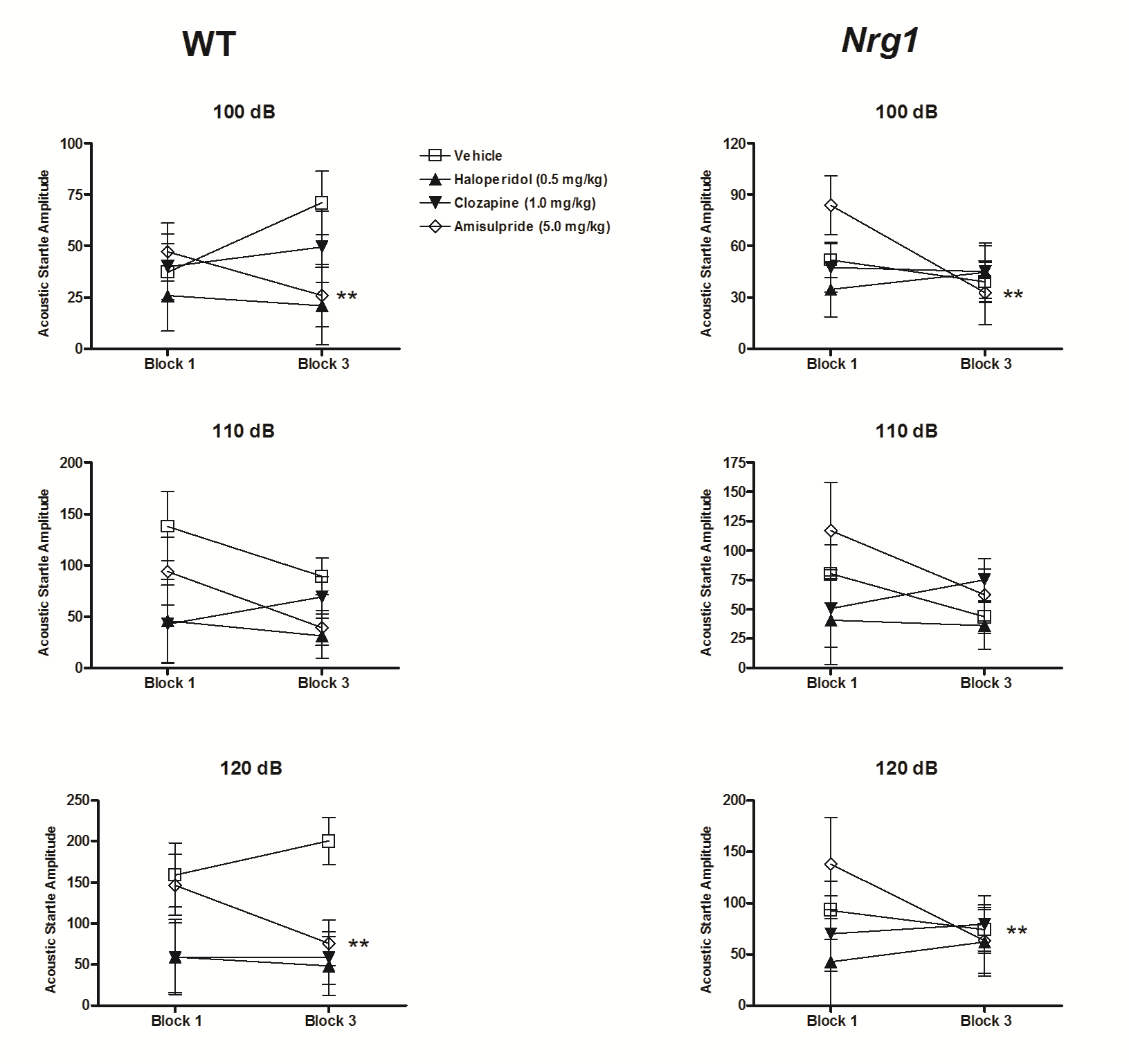


Supplementary Figure 2: Habituation to the acoustic startle response (ASR; arbitrary units) to a 100, 110, and 120 dB pulse in WT and *Nrg1* mice across four treatment conditions (vehicle, haloperidol (0.5 mg/kg), clozapine (1.0 mg/kg), amisulpride (5.0 mg/kg)) based on data collected at RCSI, Dublin. All data are presented as means ±SEM. ** *P* < 0.01 (amisulpride *vs* vehicle).

**REFERENCES**

1. Bespalov A, Jongen-Relo AL, van Gaalen M, et al. Habituation deficits induced by metabotropic glutamate receptors 2/3 receptor blockade in mice: reversal by antipsychotic drugs. *J Pharmacol Exp Ther*. 2007; 320: 944-50.

2. van den Buuse M, Gogos A. [Differential effects of antipsychotic drugs on serotonin-1A receptor-mediated disruption of prepulse inhibition.](http://www.ncbi.nlm.nih.gov/pubmed/17194799) *J Pharmacol Exp Ther*. 2007; 320: 1224-36 (2007).

3. Lubow RE, Moore AU. Latent inhibition: The effect of nonreinforced pre-exposure to the conditional stimulus. *J Comp Physiol Psychol*. 1959; 52: 415-419.

4. O'Leary C, Desbonnet L, Clarke N, et al. Phenotypic effects of maternal immune activation and early postnatal milieu in mice mutant for the schizophrenia risk gene neuregulin-1. *Neuroscience*. 2014; 277: 294-305.

5. Barbeau D, Liang JJ, Robitalille Y, Quirion R, Srivastava LK. Decreased expression of the embryonic form of the neural cell adhesion molecule in schizophrenic brains. *Proc Natl Acad Sci USA*. 1995; 92: 2785–2789.

6. Zhang R, Watson DG, Wang L, et al. Evaluation of mobile phase characteristics on three zwitterionic columns in hydrophilic interaction liquid chromatography mode for liquid chromatography-high resolution mass spectrometry based untargeted metabolite profiling of Leishmania parasites. *J Chromatogr A.* 2014;1362:168–79.

7. Zhang R, Zhang T, Ali AM, Al Washih M, Pickard B, Watson DG. Metabolomic profiling of post-mortem brain reveals changes in amino acid and glucose metabolism in mental illness compared with controls. *Comput Struct Biotechnol J*. 2016;14:106-16.

8. Kirwan GM, Johansson E, Kleemann R, et al. Building multivariate systems biology models. *Anal Chem*. 2012; 84:7064–71.

9. Benjamini Y, Hochberg Y. Controlling the False Discovery Rate a Practical and

powerful Approach to Multiple Testing. *J R Stat Soc.* 1995; 57:289–300.

10. O'Tuathaigh CM, Waddington JL. Closing the translational gap between mutant mouse models and the clinical reality of psychotic illness. *Neurosci Biobehav Rev*. 2015; 58: 19-35.

11. O’Tuathaigh CM, Babovic D, O’Sullivan GJ, et al. Phenotypic characterization of spatial cognition and social behavior in mice with ‘knockout’ of the schizophrenia risk gene neuregulin 1. *Neuroscience*. 2007; 147: 18-27.
